# Whole Body Coordination for Self-Assistance in Locomotion

**DOI:** 10.3389/fnbot.2022.883641

**Published:** 2022-06-10

**Authors:** André Seyfarth, Guoping Zhao, Henrik Jörntell

**Affiliations:** ^1^Lauflabor Locomotion Laboratory, Institute of Sport Science and Centre for Cognitive Science, Technische Universität Darmstadt, Darmstadt, Germany; ^2^Neural Basis of Sensorimotor Control, Department of Experimental Medical Science, Lund University, Lund, Sweden

**Keywords:** biomechanics, neural control, walking, swing leg, trunk, stance, body mechanics, human gait

## Abstract

The dynamics of the human body can be described by the accelerations and masses of the different body parts (e.g., legs, arm, trunk). These body parts can exhibit specific coordination patterns with each other. In human walking, we found that the swing leg cooperates with the upper body and the stance leg in different ways (e.g., in-phase and out-of-phase in vertical and horizontal directions, respectively). Such patterns of self-assistance found in human locomotion could be of advantage in robotics design, in the design of any assistive device for patients with movement impairments. It can also shed light on several unexplained infrastructural features of the CNS motor control. Self-assistance means that distributed parts of the body contribute to an overlay of functions that are required to solve the underlying motor task. To draw advantage of self-assisting effects, precise and balanced spatiotemporal patterns of muscle activation are necessary. We show that the necessary neural connectivity infrastructure to achieve such muscle control exists in abundance in the spinocerebellar circuitry. We discuss how these connectivity patterns of the spinal interneurons appear to be present already perinatally but also likely are learned. We also discuss the importance of these insights into whole body locomotion for the successful design of future assistive devices and the sense of control that they could ideally confer to the user.

## 1. Introduction

Whenever we walk, there is a noticeable mechanical coupling between the upper and the lower body (Dietz and Michel, 2009; Zehr et al., 2016). The reason that the coupling exists is obvious in quadrupeds. But why is it present also in humans and what are the properties of this coupling? There is a common idea that this is an effect of a central pattern generator (CPG) located in the neuronal circuitry of the spinal cord (Frigon, 2017). Alternatively, it can also be considered to depend on mechanical effects. The human body is structured as an arrangement of body segments connected through joints. These segments are composed of rigid components (e.g., skeleton) and soft tissues like skin, muscles, and ligaments. This indicates that any movement will cause inertial effects that potentially engage all distributed masses across the whole body and hence will be present across most movements. In biological systems, commonly used movements can be described by low-dimensional template dynamics (Full and Koditschek, 1999) encompassing movements across the whole body. This suggests that there is also an active contribution, i.e., neural control, to the coupling between the different parts of the body. Given that the distribution of inertial effects across the body will be very different between species, between individuals, and with age, as well as being dependent on the type of movement, it is likely that the neural controller must to a large extent learn and adapt to them. Several of these aspects are already known to the robotics community (Siddall et al., 2021).

A proper synchronization between the movements of the upper and lower limbs during human walking can be beneficial in terms of energetics and gait stability in locomotion. For instance, natural arm swing, which can appear quite early in humans (La Scaleia et al., 2018), can reduce whole-body vertical angular momentum and improve walking stability (Ortega et al., 2008; Collins et al., 2009). Although the natural arm swing is primarily the result of passive dynamics of the whole-body movement, the metabolic energy expenditure during walking can be significantly reduced with a natural arm swing compared to walking without an arm swing and anti-normal arm swing conditions (Collins et al., 2009).

During upright walking, the compliance of the upper body is important for self-assistance. Self-assistance means that the compliantly coupled masses of the body and the resulting inertial effects contribute to an overlay of functions that are beneficial for the brain, or a controller, to more efficiently solve the underlying motor task. This becomes evident when a parent is carrying a sleepy child. With its flexible body, the child naturally follows the upper body movements of the parent. The situation, however, becomes quite challenging when the child is angry. Then its body stiffens up and carrying it becomes much more difficult. This observation indicates the role of distributed compliance in the human body, which may support or hamper a movement. The fundamental role of body mechanics in the control of movement is often referred to as embodiment (Pfeifer et al., 2007) or intelligence by mechanics (Blickhan et al., 2007).

For biological systems to better utilize the advantages described above, we have a central nervous system (CNS). The mechanisms by which the brain generates and controls movements are still not fully clear, but it is an intriguing issue how biological systems can find resource-efficient solutions to this problem. The final stage in the nervous system for all motor commands is the spinal cord. The spinal cord contains a rich circuitry of spinal interneurons, which can house an extensive set of functions. It supports all forms of somatic motor control below the neck and likely contributes to the fact that commonly used movements, including locomotion (Grillner, 2003; Hultborn and Nielsen, 2007), can be described by low-dimensional template dynamics, or muscle synergies (Santello et al., 2013). An essential part of the function of the spinal interneuron circuitry depends on that it receives sensory feedback from a huge number of sensory afferents that transmit information to the spinal cord about the biomechanical state of the body (Hultborn, 2001; Spanne and Jörntell, 2013). These sensors enable direct spinal network responses to such state changes through local circuitry functions (sometimes ascribed to be reflexes), and also inform higher-order centers of the conditions of the external world to enable goal-directed behavior.

Understanding these principles of whole body movement has potentially major importance for the neuroscience of movement control, the design of future robotic controllers, but also the design of assistive systems for humans. As we describe in the article, because of the whole body nature of biological motor control, the body and the brain can draw advantage of a variety of self-assistance mechanisms. Such mechanisms can serve as a template for the design and control of future assistive systems.

## 2. Interaction Between Biomechanical and Neural Systems in Whole Body Control

**Figure 1:**
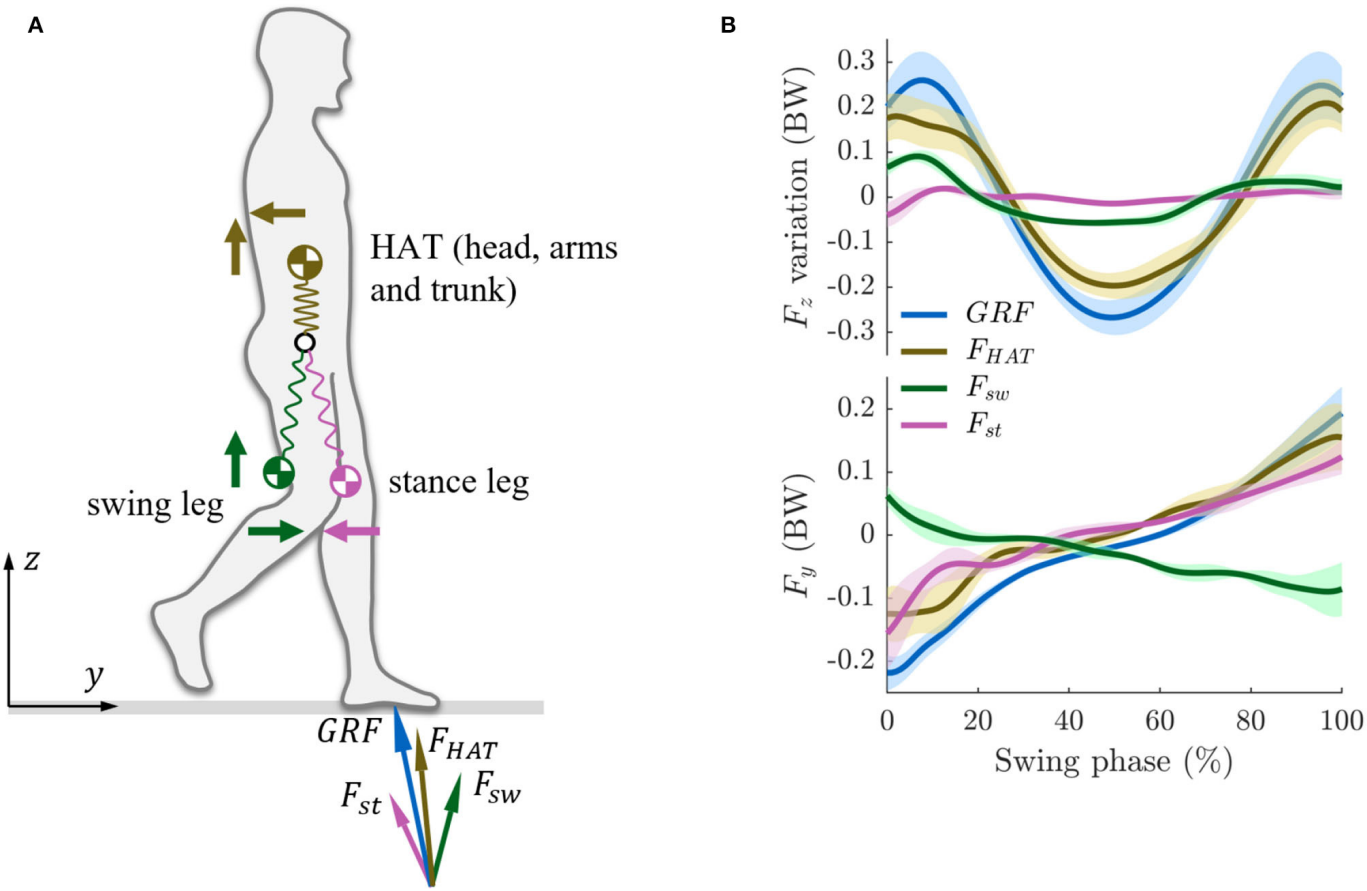
**(A)** Mechanical coupling between the upper body (head, arms, and trunk, HAT) and the lower body (swing leg and stance leg) during walking. The Ground Reaction Force (GRF) is decomposed into the vectors *F*_*HAT*_, *F*_*sw*_, and *F*_*st*_ denoting the inertial forces (the mass multiplied by the acceleration) of the HAT, swing leg, and stance leg. **(B)** *F*_*z*_ and *F*_*y*_ denote the inertial forces in vertical and horizontal (fore-aft) directions. The forces are normalized to body weight (BW). We do not show the full gait cycle, but only the swing phase as we describe the interaction between the stance leg, swing leg, and the upper body. During walking, following the swing phase, there is a double stance phase during which the upper body continues to move forward due to inertia and then triggers the next swing phase.

The required coordination of distributed body masses in the human body during locomotion can be illustrated by a simple model. Consider a set of two (or more) masses that are aligned vertically in serial order and connected with linear springs (Figure 1A). These masses could represent the leg segments, i.e., the stance leg and the swing leg, and the trunk, i.e., the upper body including the head, arms, and trunk (HAT) (Ahmad Sharbafi and Seyfarth, 2017). For example, during walking, we cyclically move the masses up and down. Even if the values of the spring stiffnesses are close to perfectly tuned, the masses will soon start to move out-of-phase due to inertia and the dynamics of the system (Sarmadi et al., 2019). In biology, such mechanical interference can be a serious problem for efficient movement control. To illustrate this principle by an example from civil engineering, consider walking on a flexible bridge with a resonant frequency that is close to the resonant frequency of your own body during walking. This situation can be very difficult to control, and thereby unpleasant, as was the case at the opening of the Millenium Bridge in London in the year 2000. Here, the lateral oscillations of the bridge were caused by the synchronization of the individual walking patterns. This was an undesired synchronization that required closing and redesigning the bridge.

In order to keep the movement of the masses synchronized, a small amount of damping could be added to the springs. This is in fact an effect created by the functional damping of muscles (Haeufle et al., 2010). But damping could result in the initial oscillation will soon become reduced in amplitude. To keep the movement ongoing, the damping needs to be compensated by active muscle control: By changing the activation signal, the spatiotemporal pattern of muscle forces generated by the CNS can be modulated such that the musculoskeletal system remains in an ongoing movement, such as described in vertical human hopping (Geyer et al., 2003; Schumacher and Seyfarth, 2017). For cyclic movements, these neuromechanical pattern generators require a proper tuning by the neural system which can result in coordinated movements of the different parts of the body, as observed in locomotion. In fact, without such active contribution to the dampening control, due to the mechanical interference, it is not possible to succeed in having two or more spring-masses if they are coupled to the same base of reference (as is the case with the insertion of the vertebral column into the hip).

In biological systems, the CNS can achieve this dampening control by learning when to activate which muscles to what appropriate degree. To fulfill this task, the CNS needs to monitor in which phase of the cyclical movement we are currently in, and update the patterns of muscle activation accordingly. The gateway into the CNS to monitor that phase, or indeed to monitor any biomechanical effects created by the muscle output patterns, are all the sensors distributed across the body. Such sensor information reaches the spinal cord and will inevitably affect the state of the network of spinal interneurons (Hultborn, 2001; Spanne and Jörntell, 2013) and, therefore, become a crucial component of the neuromechanical pattern generator. Put in more abstract terms, there is always an optimal neuromechanical pattern for fast running, jumping, walking, etc, that the CNS can approximate through learning (Cheung et al., 2020). A useful design of the neural system would be such that these neuromechanical activity patterns, or functions, can be approximated sufficiently well at the smallest possible cost in terms of the neuronal circuitry infrastructure. If the system strives to use a minimum of its neuronal network capacity for each function we want to represent, the CNS could achieve to represent as many movement patterns or movement parameters as possible with the limited resources that the CNS has. The spinal interneurons are also extensively interconnected with the cerebellum (Clendenin et al., 1974; Geborek et al., 2013b, 2014; Spanne and Jörntell, 2013; Jörntell, 2017; Mogensen et al., 2017), which may be important for learning and superimposing other movement components or parameters on the movement patterns to achieve a higher level of control. One example would be to maintain balance, which is a dominant continual challenge in nature where the smooth horizontal ground, which often is used in the lab environment, is rarely present. Therefore, under more natural circumstances of biological locomotion, information from the balance organ of the inner ear is one example of a function that needs to be continually taken into account in the neuromechanical pattern generator.

## 3. Biomechanical Considerations

When the body moves, there will be a coupling of inertial forces across the different body masses. A body mass can be defined as the mass of an arbitrarily defined segment of the body. Investigating how the upper body (HAT), the swing leg, and the stance leg interact and coordinate with each other can help us further understand the coupling mechanisms between different body masses/body parts during walking. A key dynamic feature in human walking is that the swing leg moves in phase with the upper body in the vertical direction, but out of phase with it in the horizontal direction (Zhao and Seyfarth, 2016). Surprisingly, there are not a lot of inertial dynamics in the stance leg. These arrangements come with several functional advantages: they help produce inertial vertical forces (resulting in the Ground Reaction Forces, GRF), they reduce the risk of slipping on the ground, and they make the locomotion speed more steady and, thus, help conserve energy (Zhao and Seyfarth, 2016).

Figure 1B illustrates the contributions to the total GRF of the HAT, the swing leg, and the stance leg motions during the single-support phase of walking (i.e., when the swing leg is in the air). The swing leg and the HAT create in-phase M-shape force patterns in the vertical direction (*F*_*z*_ in Figure 1B). Together with the fact that the swing leg needs to be swung forward, this is implying that there is a series of inertial forces (load) throughout the body. The muscles will need to react to these forces in an appropriate manner in order to achieve the necessary damping. The swing leg contributes about 25% to the M-shape pattern of the vertical component of the GRF (Figure 1B). The vertical force generated by the stance leg does not have a pronounced M-shape pattern in the vertical direction (in fact, the force is almost constant), which implies that it hardly contributes to the walking dynamics of the system. In the fore-aft direction (walking direction), the swing leg creates an out-phase force pattern while the stance leg creates an in-phase force pattern with respect to the HAT force (*F*_*y*_ in Figure 1B). The swing leg and the stance leg force in the fore-aft direction hence canceling each other out. The resulting reduction in horizontal forces reduces the risk of slipping. Hence, the results indicate that the swing leg motion does not impair but supports the gait dynamics.

These findings indicate what underlying structure of the biomechanical gait templates would be required for efficient walking. Through these templates, the dynamics of the human body, which inherently has a very high number of degrees of freedom, can be mapped to a low-dimensional model with only few functional parameters (Maus and Seyfarth, 2014). In the stance leg, which has an extended knee angle, the leg stiffness originates not only from the muscles in the stance leg but also significantly from a concerted action of the swing leg and the upper body. Here, the composition can be different depending on the direction (e.g., vertical vs. horizontal forces). The swing leg contributes to the overall “leg stiffness” (Song et al., 2016) represented in the spring-loaded inverted pendulum (SLIP) model to describe the center of mass (COM) of the body during locomotion (Geyer et al., 2006) and to verticalization of ground reaction forces as described in the virtual pivot point (VPP) model (Maus et al., 2010) explaining the requirements to maintain postural balance (i.e., keeping the body aligned vertically during locomotion). Thus, it serves as an “assistive system” to achieve the locomotor subfunctions (stance and balance) (Ahmad Sharbafi and Seyfarth, 2017).

## 4. Neural Considerations

Hence, the added flexibility that comes with a soft body, creates inertial effects that, if appropriately controlled through muscle damping, can be advantageous for efficient locomotion. In biological systems, the brain must actively control the distribution of these inertial forces using adequately timed patterns of muscle contraction.

Muscle contraction is directly controlled by the alpha-motorneurons of the spinal cord. The alpha-motorneurons are in turn controlled by a large network of spinal interneurons. The spinal interneuron network has a yet not well understood complexity of potential functional properties. Whereas, voluntary motor command always emanates from the CNS above the spinal cord, the planned actions have to be executed *via* the spinal interneuronal network. This network is often in textbooks associated with “reflexes” and simple input-output relationships. More recent analyses and theories however indicate that it in fact is likely to conduct much more integrated sensorimotor functions (Santello et al., 2013). For example, the body contains the order of 1,00,000's mechanosensors, each one of which communicates its information into the spinal cord network (Hultborn, 2001). Combined with the fact that spinal interneurons are extensively connected to each other, this system contains the necessary ingredients to enable rich sensorimotor dynamics. It has been shown that as long as there is excitatory input to spinal cord systems (in different animals: lamprey, cat, rat, human), they can produce highly complex sequences of muscle activation patterns, including locomotion itself (Duysens and Van de Crommert, 1998).

**Figure 2:**
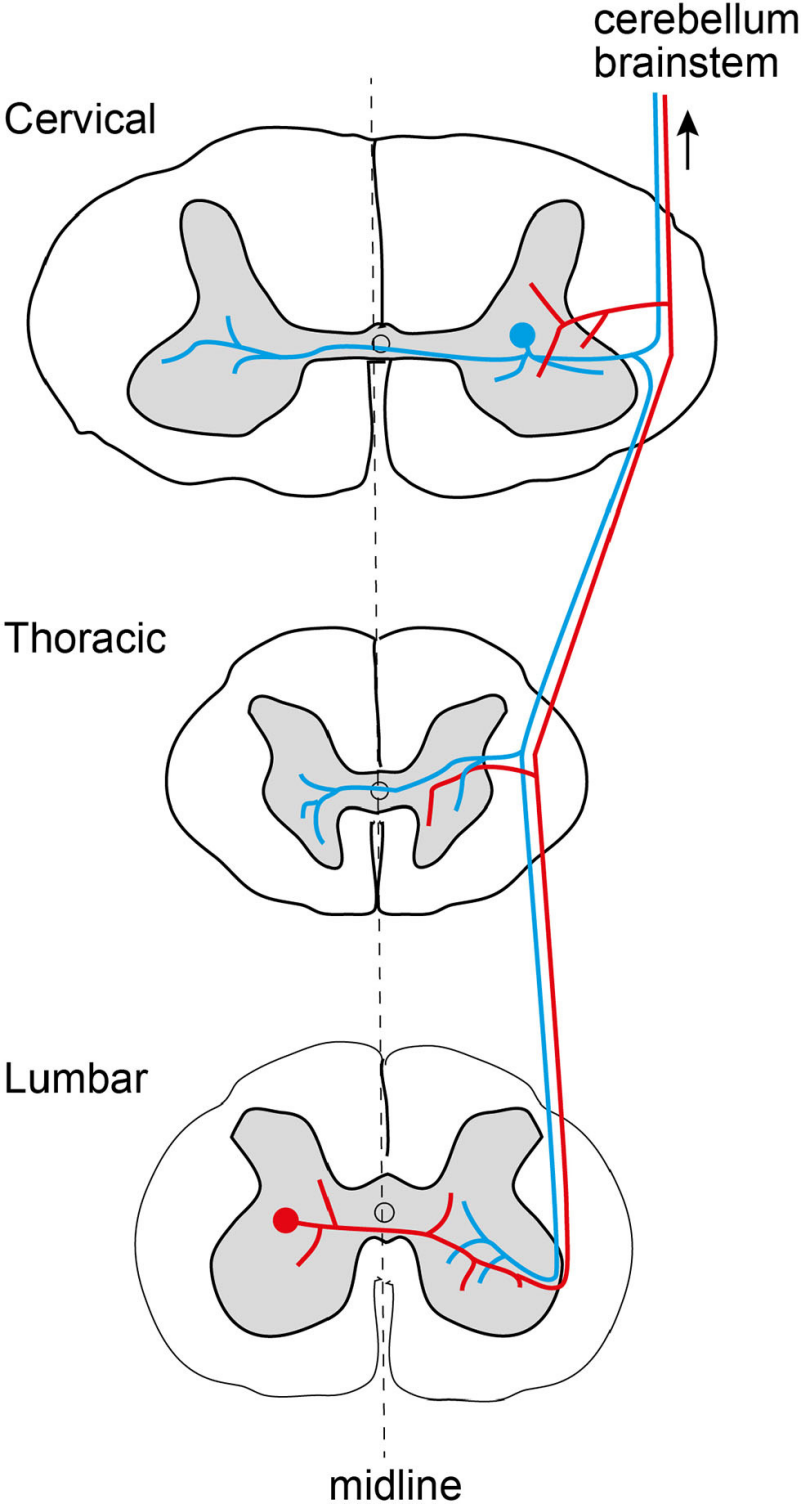
Existence of individual spinal interneurons (red and blue) with extremely divergent axons, spanning multiple spinal cord segments, for both upper and lower limbs, uni- or bilaterally, as well as trunk muscles. In this example, one interneuron has its cell body in segment C7 (blue, upper body), and one interneuron has its cell body in segment L5 (red, lower body).

In order to understand the dynamics that the spinal cord system can provide, it is necessary to understand its infrastructure. An important aspect of this neuronal network infrastructure is the degree and extent of interconnections between the different neurons of this network (Figure 2). Traditionally, spinal interneurons have been understood to be segmental, i.e., they have their dendrites and axons confined to be within one segment of the spinal cord. Given only these connectivities, each segment could be a relatively isolated functional unit. However, the presence of intersegmental interneurons was discovered a relatively long time ago, and more recently the function of longer-range propriospinal interneurons has been revealed as important for integrated arm and hand movements (Alstermark and Isa, 2012; Kinoshita et al., 2012). Even though these neurons were still primarily explored for their contributions to muscle activation within isolated limbs, spinal interneurons can have much more far reaching interconnections than that. For example, individual spinal interneurons can span many or all segments of the spinal cord (Hirai et al., 1978; Ekerot, 1990; Grottel et al., 1998; Mrówczyński et al., 2001; Krutki and Mrówczyñski, 2002; Krutki and Mwrowczynski, 2004) and they are hence in a position to contribute to the coordination of whole body movements (Reed and Magnuson, 2013), which are present already around birth (Beliez et al., 2015). These neurons have very long axons, which can traverse the whole longitudinal extent, bilaterally (Geborek et al., 2014), of the spinal cord where they send off collaterals that innervate other spinal interneurons in addition to sending projections to the cerebellum and the brainstem (Mrówczyński et al., 2001; Krutki and Mrówczyñski, 2002; Krutki and Mwrowczynski, 2004; Geborek et al., 2013a). Hence, network-wise it seems clear that even individual spinal interneurons have connectivity that would enable them to coordinate muscle activation patterns across several limbs and the trunk.

These anatomical observations of the infrastructure of the spinal cord neuronal network were all made in quadrupeds. However, observations on arm reflexes during locomotion in humans suggest that such multi-segment coordination of spinal cord activity occurs also in bipeds (Zehr and Haridas, 2003; Pearcey and Zehr, 2019). Specifically, nerve stimulation of the arm can give different impacts on leg motor control depending on which part of the step cycle it was delivered, and the effects occurred so rapidly that they had to involve local spinal and/or spinocerebellar effects (Haridas and Zehr, 2003).

These findings on the network infrastructure in the spinal cord suggest that a rich sensory interaction between the different parts of the body is provided which could be the basis for the CNS to control the observed concerted action of the upper body and the swing leg, both supporting the stance leg to generate the required forces counteracting gravity and to keep the body orientation balanced with respect to gravity. The biomechanical gait templates (e.g., SLIP model for walking and running, VPP model for postural balance) and matching neuromuscular control models (e.g., force-feedback based control of leg muscles) can provide the basis for a network integrating sensory signals (e.g., the ground reaction force) to generate specific spatio-temporal patterns of muscle activation across different parts of the body. It remains for further investigation, whether these inter-body coordination networks will follow similar neuromuscular design rules as found for creating bouncing leg function (Geyer et al., 2003) and postural control (Davoodi et al., 2019).

Hence, as described above, the spinal neuronal network already before birth contains the necessary infrastructure to achieve coordinated activation patterns across the entire spinal cord and, therefore, across all the muscles of the body. However, there still remains the challenge to get all these neuronal connections shaped to fulfill the task of adequately controlling the inertial forces caused by whole body movements. Moreover, the ontogenetic organizational plan that defines the network infrastructure during embryonic development generates the same set of spinal cord neuron types across a vast array of species (Grillner, 2018; Jung et al., 2018), e.g., the mouse, the whale, and the primate. These species obviously have bodies of very different biomechanical properties, different movement patterns, and consequently, they require different neuromechanical pattern generators. This is hard to explain without assuming that the spinal cord circuitry ‘phenotype’ is to a large extent shaped by the spinal neuronal network learns plant biomechanics as an essential part of its early development (Kohler et al., 2020; Enander et al., 2021, 2022). This can be achieved by a neural network that initially is a passive follower to the anatomically predefined biomechanical dynamics and corresponding sensor activation patterns during locomotion. If the body anatomy is correctly configured, locomotion will be the natural output of the body, much as in passive dynamic walker systems (Collins et al., 2005). But once learned, the network can then be well positioned to take active control when more challenging control conditions create a need thereof. The entrainment of the spinal cord circuitry to the plant mechanics is presumably a first critical step for enabling subsequent voluntary control of plant movements, to enable larger versatility of movement, greater adaptability, and higher precision with respect to several major low-level problems, such as maintaining balance under challenging conditions.

## 5. Discussion

In the present review, we wanted to raise awareness of the yet relatively unexplored role of the upper body in bipedal locomotion and to highlight that there is an abundance of open research issues within this subject area. By examining the neural infrastructure that could underlie such control, we moreover found that there is ample substrate for the concept of 'whole body motor control', relevant for bipedal and quadrupedal locomotion alike. Biomechanical considerations moreover point to the fact that there are inevitably body-wide mechanical couplings that the controlling neuronal circuitry needs to take into account, and in fact can use to its advantage to achieve more efficient movement control.

The main insight that we highlighted is that the whole body, including the HAT, is substantially contributing to the spring-like body dynamics. By shifting the “compliant” (stance) leg function to the remaining body, the stance leg can operate in a more straight/extended configuration, which requires less work to compensate for gravity. For instance, the major leg extensor muscles are turned off during mid-stance in walking (Van Hedel et al., 2006). Hence, during walking the upper body must use appropriate muscle activation patterns to achieve tunable compliance/damping to get in resonance with the vertical forces that inevitably will occur during the walking cycle. We moreover pointed to the assistive role of the swing leg and the upper body to achieve an advantageous center of mass dynamics. Antagonist action of swing leg to counter-act horizontal leg force generation is highly useful to reduce external acceleration and deceleration work, which in turn results in less risk of slipping on slippery ground. Slipping can be expected to be one of the main problems that need to be solved in locomotion in nature, especially if there are changing ground conditions. But in animals, overt slipping is typically a rare event, which could be due to that the CNS has devoted large efforts to prevent slipping. One aspect of future studies should be to look into information extraction from the skin of the foot sole, which can “predict” imminent slip, wherein the spinocerebellar system likely also play a crucial role.

Open questions are how and to what extent the neural system utilizes these potential assistive functions that arise due to biomechanical effects. We pointed out the existence of individual spinal interneurons that have branching patterns that would enable them to achieve coordinated muscle activation patterns across the trunk, the upper, and the lower body. However, appropriately timed muscle activation patterns will require additional contributions from more local spinal interneuron circuitries. Optimizing the utilization of the assistive functions provided by the biomechanics may in addition require contribution from the cerebellum, which can be thought of as a coprocessor capable of extending the functionality of the spinal cord circuitry (Jörntell, 2017), as well as other motor control systems. But there are many more research issues left to explore in relation to this subject area. Is there a locomotor synergy pattern in the upper limbs that all people will show? Or are they individualized to some extent? How does it depend on the state, such as emotional and/or motivational state, which is signaled across neurons distributed in the entire CNS (Allen et al., 2019).

### 5.1. Outlook: Transfer to Robotics and Assistive Devices

The coordination between body parts would be extendable also to the coordination between bodies, including human and orthotic systems/assistive robotic prostheses. The ability of the biological system to coordinate the movements between different body parts (e.g., arms and legs Dietz and Michel, 2009), i.e., the ability to self-assist, could be a proper design template for achieving assistance between two systems (e.g., a human jumping on a trampoline). The distributed compliance across the body parts can be understood as a network of dynamic mechanical effects. This mechanical network provides a set of dynamic functions which can assist and facilitate neural control. It can act synergistically with the controlling neuronal network, whose role can then be simplified to adjusting muscle compliance across specific muscles and timepoints.

If one has a proper understanding of the self-assistive functions that the biological system utilizes, then one also is in a better position to design external systems that are concerted with those self-assistive functions. Hence, the ability of the human body to assist itself (e.g., cooperation of the upper and lower body) can be understood as a blueprint for the design and control of assistive systems. Here, one critical question is how tightly the mechanical and the sensory-motor systems of a human and a machine should be coupled in order to achieve cooperation. This includes the capacity of the assistive system to identify the individual needs and intentions for supporting the user depending on the movement task, the human motor function, and the environment. The self-assistive capacity of the human body could be used for tuning the assistive device with the help of the user. For instance, the remaining motor functionality of the upper limbs could be connected to the fine-tuning of the control of a leg prosthesis by taking advantage of the plasticity of the remaining (non-affected) neuromuscular system of the upper body. The assistive device could learn to adapt the distributed compliance across the body segments by matching the passive mechanics with the biological counterpart and by fine-tuning the mechanics with sensory feedback control schemes. Compliant actuators (e.g., pneumatic muscles) can also be implemented in robotic and assistive systems (Mohseni et al., 2020) to generate external mechanical networks with dynamics that are more similar to biology, and with which the human neural controller thereby might integrate more naturally and intuitively.

## Data Availability Statement

The raw data supporting the conclusions of this article will be made available by the authors, without undue reservation.

## Author Contributions

AS and HJ were responsible for the conceptualization of this study. AS and GZ were responsible for the biomechanical part of the study. HJ was responsible for the neural part of the study. All authors prepared the manuscript together. All authors contributed to the article and approved the submitted version.

## Funding

This study was supported by the German Science Foundation (DFG) under project numbers 446124066, 456562029, and 450821862 and the EU H2020 FETOpen project #829186 ph-coding.

## Conflict of Interest

The authors declare that the research was conducted in the absence of any commercial or financial relationships that could be construed as a potential conflict of interest.

## Publisher's Note

All claims expressed in this article are solely those of the authors and do not necessarily represent those of their affiliated organizations, or those of the publisher, the editors and the reviewers. Any product that may be evaluated in this article, or claim that may be made by its manufacturer, is not guaranteed or endorsed by the publisher.
